# Incidence of pulp sensibility loss of anterior teeth after paramedian insertion of orthodontic mini-implants in the anterior maxilla

**DOI:** 10.1186/s13005-016-0134-9

**Published:** 2017-01-06

**Authors:** Jan Hourfar, Dirk Bister, Jörg A. Lisson, Björn Ludwig

**Affiliations:** 1 Department of Orthodontics, University of Heidelberg, Heidelberg, Germany; 2 Department of Orthodontics, Guy’s and St Thomas’ NHS Foundation Trust and King’s College Dental Institute, London, UK; 3 Department of Orthodontics, University of Saarland, Homburg/Saar, Germany; 4 Private Practice, Am Bahnhof 54, 56841 Traben-Trarbach, Germany

**Keywords:** Orthodontic mini-implant, Paramedian insertion, Maxilla, Pulp sensibility loss, Anterior teeth

## Abstract

**Background:**

The aim of this retrospective investigation was to evaluate the incidence of loss to pulp sensibility testing (PST) of maxillary front teeth after paramedian (3 to 5 mm away from the suture) orthodontic mini-implant (OMI) insertion in the anterior palate.

**Methods:**

A total of 284 patients (102 males, 182 females; mean age was 14.4 years (±8.8) years at time of OMI-Insertion) with a total of 568 OMIs (1.7 mm diameter, length 8 mm) were retrospectively investigated. A binomial regression analysis was performed to explore covariates, such as age, gender, inclination of upper central incisors, dentition status and insertion position of OMIs that could have contributed to loss of sensibility. Statistical significance was set at *p* < 0.05.

**Results:**

Loss of response to PST was encountered during retention in 3 out of 284 patients and the respective OMIs had been placed at height of the second rugae (R-2). Affected teeth were a right canine, a left lateral and a left central incisor. Subsequent root canal treatment was successful. Results of the binomial regression analysis revealed that the covariate insertion position (R-2) of OMIs (*p* = 0.008) had statistically significant influence on loss of response to PST.

**Conclusions:**

(1) Although there was no radiographic evidence for direct root injury, the proximity of the implants to the anterior teeth was nevertheless statistically related to loss of PST. (2) In all cases of PST loss OMIs were inserted at the second rugae. Therefore OMIs should be placed either more posteriorly, at the third rugae or in the median plane. (3). Loss of PST was not increased for patients with palatal OMI (0.18%) compared to samples without OMI (0.25%).

## Background

Sensibility is defined as the ability to respond to a stimulus and testing of the dental pulp, which can be performed using different techniques. In clinical practice commercially available refrigerant sprays (cold - tests) are often used for pulp sensibility testing (PST) [1] and the response is recorded as positive or negative. Various factors such as previous trauma [2], patient age [3], periodontal attachment loss [4] or medications (sedatives, tranquilizers, analgesics) [5] are known to have an influence on the response. It is known that orthodontic tooth movement can affect PST response temporarily [6], but sensibility is thought to return to normal after completion of treatment. The authors state that there is no agreement in the literature regarding potential long-term sequelae: reported pulpal responses after orthodontics included circulatory vascular stasis and necrosis [7]. Cases of pulpal necrosis following orthodontic therapy have been occasionally reported [8, 9], but this is unusual.

Adjunctive procedures such as extensive enamel stripping [10] and subtractive Odontoplasty [11, 12] may lead to a critical rise in intrapulpal temperature [10] with subsequent pulp necrosis [13]. Clinicians performing PSTs use the qualitative sensory manifestations to extrapolate the state of the pulp to assess the “vitality” of the tooth [1, 14]. “Sensibility” and “vitality” are hence often used interchangeably [1, 5], although it is well known that PST can produce false positive and false negative results for vitality.

Orthodontic mini-implants (OMIs) have changed orthodontic paradigms by broadening the spectrum of dental movements [15]. Numerous risks and complications associated with the use of OMIs have been described before and specific complications such as unintentional root damage [16, 17], if severe enough can lead to loss of sensibility and vitality.

The anterior palate is most suitable [18] as insertion site for OMIs because of high success rates [19] and ideal anatomical conditions. Palatal bone quality and quantity for safe insertion of OMIs has been well documented [20–22]. Despite these findings, unintentional root damage of a lateral incisor after paramedian OMI-Insertion in the anterior palatal vault has been reported [23].

The aim of this retrospective investigation was to evaluate incidence of response loss to PST of maxillary front teeth after paramedian OMI insertion in the anterior palate.

## Methods

### Patients and treatment protocol

#### Patients

Patients with no history of previous orthodontic treatment and need for OMI supported orthodontic biomechanics were included. All patients received treatment by a single orthodontist (B. L.) in a specialist orthodontic practice (Traben-Trarbach, Germany), including fixed orthodontic appliances with OMI placement. As previously described [24–26], two OMIs were inserted symmetrically parasagittal (3 to 5 mm away from the suture) [27] into the anterior palate for appliance attachment. OMIs were loaded two weeks after insertion, because of manufacture of the appliances attached to them.

Inclusion criteria:

Unrestored maxillary permanent front teeth without history of trauma and previous dental treatment

Exclusion criteria:

- systemic diseases/disorders
- craniofacial malformations
- chemo and/or radiotherapy during tooth development
- accidents/craniofacial trauma
- history of previous surgery requiring endotracheal intubation
- dental malformations
- severe crowding of the upper front teeth
- periodontal disease
- history of previous orthodontic treatment
- tooth agenesis (except for third molars) or tooth loss
- enamel stripping or occlusal adjustments to the upper front teeth
- medications such as sedatives, tranquilizer, analgesics

#### Skeletal anchorage

Only one type of mini-implant (1.7 mm diameter, length 8 mm) was used (OrthoEasy®, Forestadent, Pforzheim, Germany). This implant system has an anodized surface and features a self-tapping and cutting design and is made from Titanium-alloy (Ti-6Al-4 V). Following patient consultation and consent, 0.2 ml to 0.5 ml of local infiltration anaesthesia (Ultracain® D-S, Sanofi-Aventis Deutschland GmbH, Frankfurt, Germany) was used. The OMIs were inserted without soft tissue incision or pre-drilling, perpendicular to the bone surface, using a motorised dental handpiece at an insertion speed of 60 RPM. Torque limitation was 30 Ncm. All OMIs were removed at debond.

### Bonding and debonding of the fixed appliance

Bonding and removal of the fixed appliances followed a standardized protocol. Self- ligating steel Brackets (Quick®, Forestadent, Pforzheim, Germany) were indirectly bonded applying a light cure bonding material (Transbond® Supreme LV, 3 M Unitek, Monrovia, Calif., USA). A halogen light was used for curing composite material according to manufacturer instructions.

Bracket removing pliers were used for debonding. The residual adhesive on each tooth was removed with fluted tungsten carbide burs and the surface finished using silicone carbide polishers. All clean-up procedures included water-cooling.

### Pulp sensibility testing

Thermal PST (cold test) of the maxillary front teeth was performed just prior to OMI-insertion, at debond of the fixed appliance/OMI-removal and 24 month post debond. Endo-Ice® (Coltène/Whaledent Inc., Cuyahoga Falls, Ohio, USA), producing a temperature of −50 °C was used. The product was applied to the teeth using a cotton wool pad. Response was recorded as either positive or negative.

Records included full documentation for the entire treatment including appropriate radiographs.

### Diagnosis of radiographic material

All radiographs were taken with an Orthophos® XG 3 (Sirona, Bensheim, Germany).

#### Panoramic x-rays (OPGs)

OPGs were available pre-treatment (initial diagnostics) and were used for the diagnosis of bony and dental anomalies/pathologies prior to OMI-insertion.

#### Cephalometric analysis

**Fig. 1:**
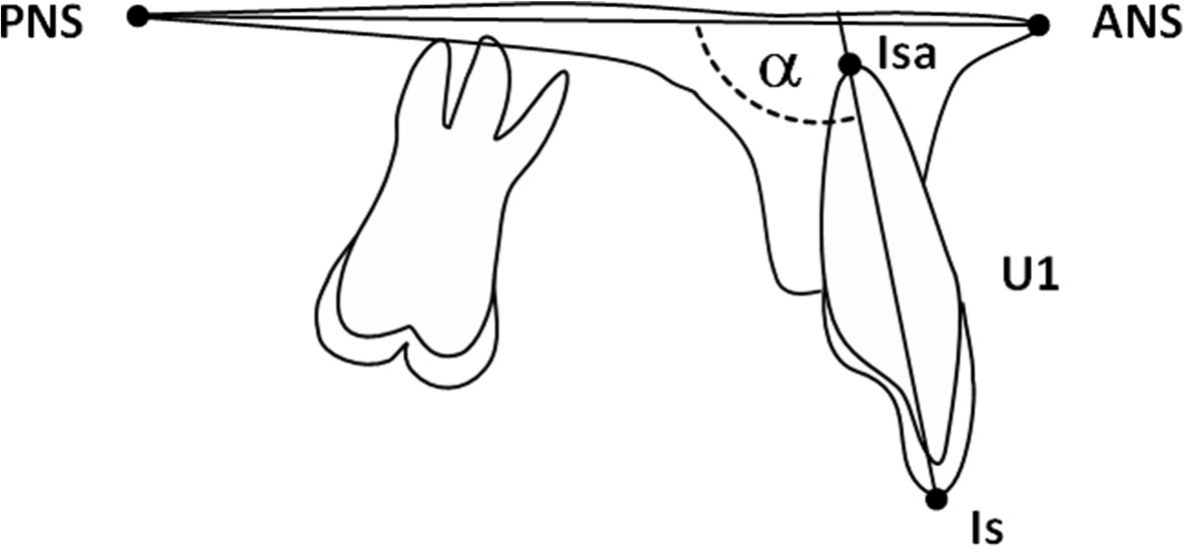
Inclination of upper central incisors. Measurement of the Inclination of upper central incisor (U1/ANS-PNS) between the palatal plane (ANS-PNS) and the long axis of U1 (Is-Isa)

Using the pre-treatment cephalograms (initial diagnostics) the inclination of upper central incisors (U1/ANS-PNS) prior to OMI-insertion was measured (Fig. 1), and 108° ± 5° [28] was regarded as a standard mean value.

### Assessment of OMIs’ insertion positions

Position of the OMIs were assessed using the plaster working models for the appliances.

Because palatal rugae have been previously described as stable, clinically visible structures [29], the insertion positions of the OMIs were classified in relation to the medial ends of palatal rugae:

1. at second rugae (R-2)
2. between second and third rugae (R-2/3)
3. at third rugae (R-3)

### Data collection and statistical analysis

Data was collated using Microsoft Excel® 2007, (Microsoft Corp., Redmond, Wash., USA). All cephalometric angular measurements and the assessment of the implant position were re-measured after three months by the same operator. Average intra-examiner reliability calculated by the coefficient of variation (COV) was 0.01 for the former and using the intraclass correlation coefficient (ICC) was 1.0 for the latter.

A binomial logistic regression analysis was performed to explore covariates, such as age, gender, Inclination of upper centrals, dentition status and insertion position of OMIs that could possibly have contributed to loss of sensibility. Statistical analysis was performed using SPSS® for Windows®, version 22.0 (IBM Corp., Armonk, New York, USA). Statistical significance was set at *p* < 0.05.

## Results

A total of 284 patients (102 males, 182 females) with a total of 568 OMIs met the inclusion criteria. All patients were of Caucasian origin. At the time of OMI-insertion the mean age was 14.4 years ± 8.8 years. 169 patients were in mixed dentition, and 109 patients were in permanent dentition. Average inclination of upper incisors (U1/ANS-PNS) was 109.81° ± 8.37°; they were hence slightly proclined. Most OMIs were inserted at the third rugae (Table 1). In none of the patients root injuries were diagnosed on the available radiographs.

**Table 1:** Distribution of OMIs insertion positions’ in relation to palatal rugae

Insertion position	Number	Percent
second rugae (R-2)	76	13.4
between second and third rugae (R-2/3)	24	4.2
third rugae (R-3)	468	82.4

**Table 2:** Details of affected patients

Gender	Patient 1	Patient 2	Patient 3
	female	female	female
Age (years)	37	11	12
Inclination U1 (degrees)	99.20	98.50	110.50
OMIs insertion positions	R-2	R-2	R-2
Affected tooth (FDI-Notation)	13	22	21

R-2, OMI’s insertion position at second rugae

### Loss of response to pulp sensibility testing (PST)

Loss of response to PST was encountered in 3 (1.06%) out of 284 patients or 0.53% per OMI. The percentage was 0.18% (*n* = 3) for the 1704 maxillary incisors and canines and 0.18% (*n* = 2) for the 1136 maxillary incisors respectively. PST was found negative in the three affected patients in the second half of two year retention phase following debond of the fixed appliances. Affected maxillary teeth were: A right canine, a left lateral and a left central incisor. Details are in Table 2.

The three affected patients initially presented with painful teeth and were hence referred to an endodontic specialist for further clinical and radiographic diagnosis. All affected teeth received root canal treatment. After successful treatment the symptoms resolved. Interestingly, no root injury was diagnosed on the intraoral films during endodontic treatment.

Results of the binomial logistic regression analysis revealed that covariates gender (*p* = 0.996), age at OMI-Insertion (*p* = 0.456), Inclination of upper incisors (U1/ANS-PNS) (*p* = 0.289) and dentition status (*p* = 0.587) had no statistically significant influence on loss of response to PST, whereas the insertion position of OMIs (*p* = 0.008) had.

## Discussion

Loss of response to PST and vitality respectively was encountered in 3 out of 284 patients of our sample. The percentage was 0.18% for the 1704 maxillary incisors and canines and 0.18% for the 1136 maxillary incisors respectively. Interestingly, these results are very similar to those of an investigation by Bauss et al. [30]. They also found a small percentage of 0.25% (*n* = 2) for 800 healthy non-traumatized permanent incisors in 200 randomly selected patients who underwent fixed treatment without OMI placement.

In all affected patients of our sample, symptoms that led to referral to an endodontic specialist were encountered during retention. Occurrence of symptoms was late, considering loss of vitality was only detected long after OMI removal and debond. However, considerable variation has been described in the literature [23, 31–33] and loss of vitality can occur up to 2 years after OMI placement [32] because root injury can remain symptomless over a long period of time. Er et al. [23] reported a periradicular lesion caused by unintentional root damage after paramedian placement of two OMIs (1.5 mm diameter, length 10 mm) in the anterior palate for a distalizing appliance in a 22-year-old female, thus requiring endodontic treatment. Two months after OMI-insertion, the patient complained of pain and the right maxillary lateral incisor was endodontically treated.

Root perforations after buccal interradicular insertion of OMIs have also been reported: two cases of maxillary first molars [32, 33] and a mandibular right lateral incisior [31]. In the latter additional periapical surgery was performed for retrograde root canal treatment.

After loss of response to PST, patients were referred to an endodontic specialist who diagnosed pulp necrosis and undertook endodontic treatment. Because no root injury could be diagnosed on the available plain film radiographs and all patients were free of symptoms after treatment, no additional cone beam computed tomography (CBCT) was performed, although this would have been the modality of choice to diagnose the exact location of the possible root injury site [34]. Therefore, we cannot completely exclude root perforations due to OMI insertion and this has to be kept in mind when considering the results of this investigation.

It was well known that OMIs did not remain stationary during orthodontics [35] and primary (direct) and secondary (migration) displacement has been observed. Primary displacement is due to the elastic characteristics of the bone whereas the latter occurs under orthodontic loading over time, caused by remodeling processes of the bone. In a systematic review by Nienkemper et al. [36] secondary displacement of OMIs was found 0.23 to 1.08 mm for the head, 0.1 to 0.5 mm for the body and 0.1 to 0.83 mm for the tip. Maximum values ranged from 1.0 to 4.1 mm for the head, 1.0 to 1.5 mm for the body and 1.0 to 1.92 mm for the tip. Tipping angles ranging from 1.0 to 2.65° were noted. The mean extrusion of OMIs ranged from 0.1 to 0.8 mm and intrusion of up to 0.5 mm was also observed. In our study OMIs were removed at the time of debond and the tip of displaced OMIs might have interfered with the tissues supplying surrounding teeth with innervation and vascularity.

Besides possible complications with the use of OMIs [16, 17] affecting PST response and pulp vitality, numerous relationships between orthodontics and adjunctive procedures respectively and the state of the dental pulp were also described [37–39]. Patients requiring adjunctive procedures to orthodontics on maxillary front teeth such as approximal enamel reduction [10] and occlusal adjustments [11, 12] were not included, because pulpal temperature may have risen critically during the procedure [10].

It has been reported that orthodontic tooth movements like intrusion might also influence PST response [7, 40]. Radiographic examination, albeit limited to two-dimensional plain film radiographs, did not reveal any close proximity between the OMIs and the roots of the teeth. It is therefore unlikely that direct Injury led to loss of vitality.

However loss of response to PST and vitality might have been caused by orthodontics itself. This may be relevant for the canine that required root canal treatment, as direct injury of this tooth during OMI-Insertion in the anterior palate was unlikely to have caused this issue and has never before been reported in literature. Moreover the patient was an adult (37 years) and older than the other patients affected (11 and 12 years). Hamersky et al. suggested [41] that orthodontic forces cause biochemical and biologic pulpal tissue changes and that orthodontic forces may be less safe as the age of the patient increases. Open apices allow vessels to enter the pulp and the increased amount of loose connective tissue in this apical area may help to maintaining pulpal blood flow during orthodontic force application; this argument has also been made by other authors [42, 43]. Remarkably Ingle et al. [44] found that the maxillary canine, which is generally not affected by dental trauma, appears to be the tooth most susceptible to pulp hemorrhage and necrosis when exposed to orthodontic force application, suggesting ischemic infarction as most likely cause.

The possibility of dental trauma before, during and subsequent to orthodontic treatment plays an important role when interpreting the results of our study. Incidence of dental trauma is subject to continuous investigation [45–47] and data from the United States revealed that 25% of the population from 6 to 50 years of age may have suffered dental trauma to the anterior teeth [45]. Surprisingly, some patients are unaware of this and many choose not to seek dental treatment [45, 48] and taking a past dental history is likely to be unreliable. Most dental injuries occur during the first two decades of life; the most accident-prone time was found from the age 8 to 12 years [46, 48]. Dental trauma is more frequent in boys than girls however there is considerable variation [47]. Maxillary central incisors, followed by the lateral incisors are most frequently involved [49]. One investigation evaluated pulp vitality in teeth suffering trauma during orthodontic therapy; prevalence of pulp necrosis was 18.6% [30] and this was much higher than our findings.

We excluded patients who had a past history of general anaesthesia; dental trauma during endotracheal intubation anaesthesia is one of the most common encountered adverse events of general anaesthesia [50]. Maxillary central incisors are affected most frequently [51]. Incidence of dental trauma reporting was found to be smaller than 0.2% [52] when assessed by anaesthesiologists compared to 12.1% [53] when assessed by dentists. It was suggested that examinations should hence be conducted by dental surgeons [54].

Alomari et al. [6] examined PST response using electric pulp testing (EPT) during and after orthodontic treatment. The threshold of response to PST using EPT was found to vary during but returned to pre-treatment values towards the end of the retention phase. The authors suggested that responses to electrical pulp testing, should be interpreted with caution during orthodontics and that a negative PST response does not always indicate pulpal necrosis.

In daily clinical practice a refrigerant spray (RS) is often used for practical reasons [55] and in our study we also used a RS producing a local temperature of -50 °C. There is little evidence which cold delivery method is most accurate in determining pulp responsiveness. Jones et al. [56] compared carbon dioxide dry ice sticks (CO2) with RS and concluded that RS and CO2 were equivalent in determining pulpal responsiveness, but the response elicited from the refrigerant spray (RS) was faster.

To distinguish between “sensibility” and “vitality” advanced techniques such as Laser-Doppler techniques (Laser Doppler Flowmetry - LDF) to measure intrapulpal blood flow can be used [1, 5]. LDF was found to be a reliable method. However, it is technique-sensitive [57] and extra-pulpal blood flow, mainly from the periodontal ligament, may contaminate the signal [58]. Moreover LDF is time-consuming [57, 59] and hence not always practical for routine clinical use.

Regression analysis showed that only the insertion position of the OMIs was a statistically significant covariate (*p* = 0.008) for loss of vitality. In the three patients affected by a negative response to cold testing the OMIs were inserted at the second rugae (R-2), and we must assume that a more posterior insertion directed to the third rugae (R-3) is more likely to preserve vitality. This is in agreement with a recent investigation by Hourfar et al. [21] which examined bone availability for OMI insertion in relation to the palatal rugae. Most bone was found in the vertical dimension at the first and second rugae for 8 mm long OMIs. Yet they stated that it was challenging to access the area of the second rugae clinically: OMIs would have to be inserted vertically to avoid damage to the incisor roots and perpendicular insertion to the bone surface might not be suitable for this area.

The inclination of upper incisors (U1/ANS-PNS) was not a statistically significant covariate (*p* = 0.289) although a slight tendency towards proclination was noted within in the sample.

To our knowledge, this study is the first retrospective study that investigates the relationship between OMI positioning and loss of PST response and pulp vitality.

Time delay of endodontic complications was accounted for in our study design by investigating the patients 24 months after debond. We propose further research using prospective designs to verify the outcome of our investigation.

## Conclusions

- Although there was no radiographic evidence of OMI induced trauma to the teeth that lost vitality, the proximity of the implants to the anterior teeth was positively and related to loss of PST (*p* = 0.008).
- In all cases of PST loss OMIs were inserted at the second rugae (R-2) and we therefore we recommend that OMIs should be placed either more posteriorly, at the third rugae (R-3), or in the median plane. This will decrease risk of trauma to the roots of the anterior teeth.
- PST/vitality loss post OMI-insertion in the anterior palate was only 0.18%. We conclude that the risk of palatal OMIs leading to loss of PST/vitality of the upper front teeth is small.
